# Evidence of circadian rhythms in non-photosynthetic bacteria?

**DOI:** 10.1186/1740-3391-8-8

**Published:** 2010-09-16

**Authors:** María I Soriano, Begoña Roibás, Ana B García, Manuel Espinosa-Urgel

**Affiliations:** 1 Department of Environmental Protection, Estación Experimental del Zaidín, CSIC, 18008 Granada, Spain

## Abstract

Examples of circadian rhythms have been described in eukaryotic organisms and in photosynthetic bacteria, but direct proof of their existence in other prokaryotes is limited and has been largely ignored. The aim of this article is to review existing evidence and to present preliminary results that suggest that the heterotrophic bacterium *Pseudomonas putida *shows regular variations in its growth pattern synchronized with light/darkness cycles. We put forward the hypothesis that circadian regulation of certain processes can take place in non-photosynthetic prokaryotes and may represent an adaptative advantage in specific environments.

## Background

Activities and physiological processes taking place with a periodicity of around 24 h have been described in many organisms [1]. Circadian cycles, synchronized and entrained by light and darkness cycles determine in mammals the periods of sleep and vigil, changes in body temperature or hormone production, among other functions. In plants, the release of hormones such as ethylene, certain developmental processes, or the release of seed and root exudates are subject to this periodicity [2]. The absence of circadian cycles is generally assumed for bacteria, except in the case of cyanobacteria, in which photosynthesis and nitrogen fixation are either spatially segregated in heterocyst-forming bacteria, or temporally separated and controlled by circadian periodicity. Both processes would not be possible at the same time in a single cell, because oxygen released during photosynthesis inhibits nitrogenase activity. Circadian rhythms in photosynthetic bacteria are being thoroughly studied, and the molecular mechanisms that allow the functioning and maintenance of the circadian clock are fairly well understood [3]. The clock is composed of three proteins, KaiA, KaiB, and KaiC, which function as a central oscillator through phosphorylation/dephosphorylation cycles. The system is synchronized via a signal input pathway in which sensory proteins are known to transmit light/darkness information to the clock, while a series of clock-controlled regulators transduce the temporal information to downstream processes. Expression of a significant number of genes appears to be modified in response to light/darkness cycles and circadian oscillations, indicating that this type of control is not limited to certain specific features of cyanobacteria. Such broader impact on different physiological processes makes us question the premise of circadian rhythms being circumscribed to this particular group within prokaryotes.

## Changing concepts of the bacterial world

The presumed lack of circadian periodicity in the physiology of heterotrophic bacteria was originally founded not on actual experimental evidence but on premises that for a long time were accepted in microbiology. One rests on the classical view of bacteria as simple reproductive machines that merely multiply as long as nutrients are available, and therefore would have no use for a regular, nearly anticipatory control system. However, the idea of a bacterial population as a mass of rudimentary organisms growing as fast as resources allow has long given way to the appreciation of a far more complex picture. It includes finely tuned sensory and regulatory mechanisms, or the ability to coordinate responses at the population level through the synthesis and release of signal molecules, in the process known as quorum sensing. A second argument is that the need of bacteria to quickly respond to external stimuli (nutrients, stressors, or sudden environmental changes) would not be compatible with circadian rhythms, which would impose fixed temporal responses. This might be true if circadian regulation were of such a strict nature that it limited the organism's adaptability to changing situations. But the existence of a circadian clock does not impede cyanobacteria, for example, to have efficient stress response mechanisms. Furthermore, it is possible to envision environmental situations where a circadian control of certain processes could be beneficial. It might be the case of plant-associated bacterial populations, given that the amount and composition of nutrients released through roots or leaves in the form of exudates varies with circadian periodicity. A mechanism for sensing light and temperature changes associated to day/night cycles, and acting as a metabolic switch to anticipate the alteration of incoming resources, could be an advantage for bacteria colonizing plant surfaces.

## Old and new evidence

A series of experiments published in 1930 by Rogers and Greenbank [4] demonstrated the existence of oscillations in the growth rate of *Escherichia coli *in fluid medium. The authors designed a 15 meter-long hollow glass spiral that was filled with liquid medium with a chromogenic indicator of lactose utilization and inoculated at one end with an *E. coli *culture. Progression of the culture was then followed over a period of several days by observing turbidity and color change and recorded by photographing the spiral at fixed intervals. Data showed periodic alternation of fast and slow growth, with peaks of fast growth appearing every 20-22 h. The authors suggested that periods of exposure to intense light could be influencing this phenomenon. However, these results were disregarded based on observations published by other researchers [5], which were actually made under different experimental conditions, during a much shorter period of time, and with other microorganisms. Later reports of circadian oscillations in the growth of *Escherichia coli *or *Klebsiella pneumoniae *exist in the literature (reviewed in [6]), but the prevailing view at the time those observations were published was that circadian rhythms were to be found exclusively in multicellular eukaryotic organisms. The discovery of this phenomenon in photosynthetic prokaryotes and unicellular eukaryotes did not bring those studies back to light.

Scattered evidence that may add weight to those initial observations can be found in more recent literature. We have already mentioned plants as bacterial habitats where circadian regulation would make sense. In fact, variations in plant-colonizing bacterial populations depending on the time of day at which samples were taken have been reported [7], and the potential role of light and plant circadian rhythms in plant pathogenesis has been recently put forward [8]. Furthermore, photoperiod can influence the response of pepper plants to the positive action of plant growth-promoting *Bacillus amyloliquefaciens *bacteria [9], so that root and shoot biomass are increased in the presence of these microorganisms only in plants under long day (12 h of light) conditions, but not under short day (8 h) conditions.

## Bacterial responses to light

While the above mentioned data have for the most part been interpreted from the point of view of plant responses, alterations in microbial physiology as a reaction to photoperiod should not be discarded. With the ever-increasing data obtained from genome sequencing projects, it has become apparent that genes encoding proteins similar to phytochromes, which in other organisms sense light and may participate in the adjustment of circadian rhythms, are present in the genomes of different bacteria. Proteins containing a light, oxygen or voltage (LOV) sensing domain are also widespread in many organisms, including bacteria. Although their function in many cases remains to be determined, a protein containing this domain has recently been shown to respond to light and to participate in stress responses in the soil bacterium *Bacillus subtilis *[10]. A similar blue light activated protein determines survival of the pathogen *Brucella abortus *within macrophages [11]; infection by this bacterium is stimulated by light, and this stimulation is dependent on the LOV protein.

It is also worth noting that proteins related to KaiC, the central oscillator of the circadian clock of cyanobacteria, can be found in other microorganisms [12]. Although their function is yet to be defined, these proteins may participate in signal transduction in some organisms. Bioinformatics analyses using BLAST programs http://blast.ncbi.nlm.nih.gov/ indicate that genes encoding phytochrome-related proteins and a putative *kaiC *homolog can be found in the genome of *Pseudomonas putida *KT2440, for example. In this strain, the protein showing similarity with KaiC appears to be part of a three-component system, along with a sensory histidine kinase protein and a response regulator, suggesting it is involved in the response of *P. putida *to environmental signal(s).

## Light/darkness cycles influence surface growth of *Pseudomonas putida*

**Figure 1:**
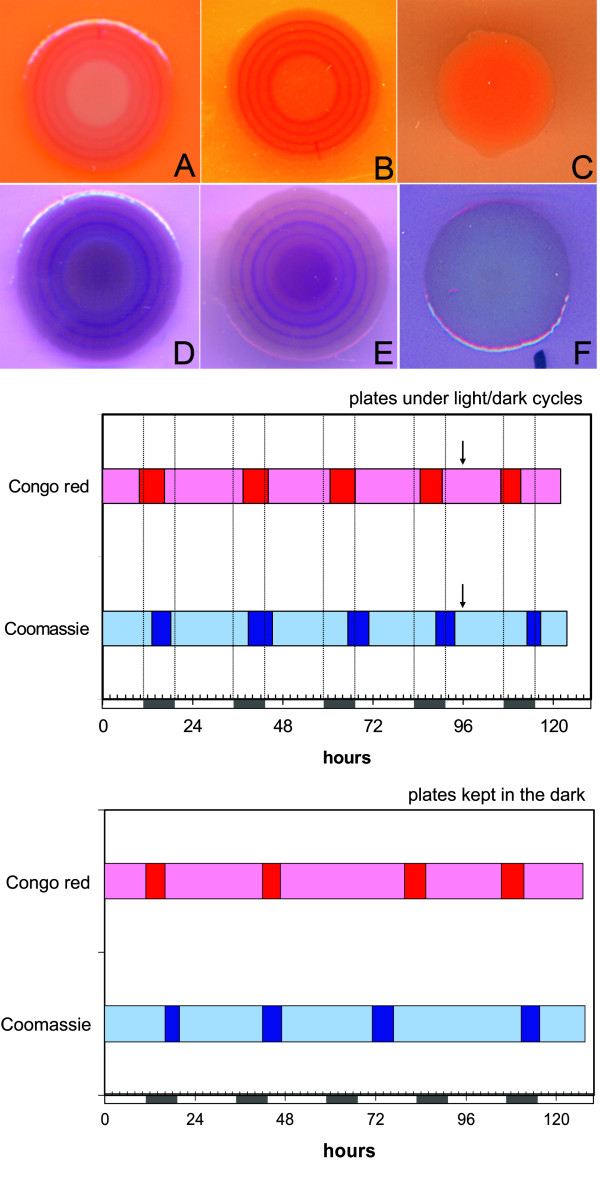
***Pseudomonas putida *displays cyclic surface variations during growth**. Plates contained LB medium (triptone 1%, yeast extract 0.5%, NaCl 0.5%, with 1% agar and supplied with 5 μm FeCl_3_) with Congo red (panels A-C) or Coomassie Brilliant Blue (panels D-F). Panels A, B, D, E correspond to patches grown under alternating light/darkness cycles (A and D, top view; B and E, bottom view; contrast has been enhanced for clarity). Panels C and F correspond to patches grown in constant darkness. The graphs show an extrapolation of the occurrence and duration of the differently colored rings in plates under cyclic light/darkness periods (top) or kept in the dark under the same conditions (bottom). Data are based on their width and the calculated growth rate for each patch (μm/h). Light and darkness periods are indicated below the X axis as white and grey bars, respectively. Arrow indicates when plates were removed from the cyclic light/darkness regime.

We have conducted a preliminary exploration of how alternating light/darkness cycles affect the growth pattern of the heterotrophic, plant-colonizing bacterium *Pseudomonas putida *KT2440. Cultures of this strain grown overnight in liquid medium were spotted on the center of Petri dishes containing rich medium with 1% agar and either Coomassie brilliant blue or Congo red (50 μg/ml). These dyes bind proteins and other extracellular polymeric substances, respectively, and allow direct visualization of surface changes appearing during colony growth. Plates were incubated for 16h in the dark, sealed with Parafilm to avoid excessive desiccation and transferred to a growth chamber with artificial illumination (white, broad spectrum compact fluorescent lamps with a CRI ≈ 82, 3,500K; E_v _≈ 32,000 lux), set at light/darkness cycles of 16 h/8 h, under constant temperature (30 ± 0.3°C). Control plates were kept in the same conditions but wrapped in aluminum foil. In a second set of experiments, plates were incubated under constant illumination. Growth and patch morphology were followed for 4 days. The emergence of concentric, regularly alternating rings with different color intensity could be observed in colonies under daily illumination/darkness cycles (Figure 1). Similar results were obtained under light/darkness cycles of 12 h/12 h (not shown). Digital images of the plates were analyzed using the ImageJ free software (Rasband, W. S., ImageJ, National Institutes of Health, Bethesda, Maryland, USA, http://rsb.info.nih.gov/ij/). After determining colony growth rate (between 34 and 37 μm/h), measurements of distances between rings were used to extrapolate the periodicity in which darker and lighter rings appeared. Successive rounds of one dark plus one light rings tend to synchronize into cycles of approximately 24 h (24.1 ± 2.5 h for Congo red plates and 24.9 ± 1.24 h for Coomassie plates; Figure 1). This periodicity was maintained for two days after the plates were removed from the alternating light/darkness conditions and then lost, although by that time agar plates were too dry to obtain sound information. Patches incubated in the dark generally showed an even appearance (Figure 1), although faint concentric patterns could be discerned in some experiments. However, when these appeared, they only approached 24 h cycles in the first two days of incubation. Afterwards, this circadian periodicity was lost, tending to irregular oscillations of the alternating colored rings. Patches under constant illumination never showed concentric growth patterns. Although the effect of temperature changes (± 1.5°C) associated with illumination regimes cannot be fully ruled out, these results suggest that variations in colony surface characteristics may be synchronized by alternating light/darkness phases, and perhaps they could also be indicative of processes in non-photosynthetic bacteria being subjected to circadian regulation.

## Concluding remarks

Our aim with this paper has been to put forward the potential interest of probing in detail the possibility of cyclic regulation in prokaryotes other than cyanobacteria, even though the current evidence may not be considered sufficiently solid. Probably, detailed research should be conducted with bacteria recently isolated from the environment, and not with the "domesticated" strains that have been under laboratory conditions for decades, since it is possible that in the latter this type of response is lost or at least damped, as is the case with other functions. In *Escherichia coli*, for example, the *gapC *gene is preferentially expressed in fresh water environments and is intact in environmental isolates, whereas laboratory strains still show environment-specific activation of its promoter but accumulate mutations in *gapC *rendering a truncated protein [13].

Circadian rhythms with a modulatory role on the physiology of heterotrophic bacteria would significantly change our view of certain aspects of microbial ecology and evolution, as previously mentioned for plant-associated bacterial populations. Besides, there might also be relevant biomedical implications; it is noteworthy that human body temperature and immune response show daily oscillations. Such well known fluctuations have been largely overlooked by clinical microbiologists but, could they actually represent an evolutionary adaptation to growth or virulence peaks in pathogenic bacteria? The idea has been previously hinted at [14], and may represent a potential new avenue of research.

## Competing interests

The authors declare that they have no competing interests.

## Authors' contributions

MIS and BR performed experiments. AMG and BR performed bibliographical research. MEU designed experiments, analyzed data and wrote the paper. All authors read and approved the manuscript.
